# Robot-assisted excision with intervertebral foramen endoscopy in the treatment of osteoid osteoma of femoral neck: a case report

**DOI:** 10.3389/fped.2025.1499394

**Published:** 2025-06-23

**Authors:** Jinying Lao, Gen Ba, Tianjing Liu, Enbo Wang

**Affiliations:** ^1^ Department of Pediatric Orthopaedics, Shengjing Hospital of China Medical University, Shenyang, Liaoning, China; ^2^ Department of Spine Surgery, Shengjing Hospital of China Medical University, Shenyang, Liaoning, China

**Keywords:** osteoid osteoma, femoral neck, robot-assisted, intervertebral foramen endoscopy, excision

## Abstract

**Background:**

The diaphyseal or metaphyseal of the long bone is a common location for osteoid osteoma(OO), however, the femoral neck lesion is rare. There proves an original method to treat this disease.

**Case presentation:**

An 11-year-old female, complained of a limp and right hip pain. Based on her history, and clinical and imaging examination, she was diagnosed with OO. Given that the location of the lesion is on the back of the femoral neck within the hip joint, and the local anatomical structure is close to the sciatic nerve and medial circumflex femoral artery, we performed robot-assisted excision with intervertebral foramen endoscopic.

**Conclusions:**

This innovative treatment completely and successfully eliminated her symptoms without recurrence at the last follow-up. Our research suggests that this technology can be widely applied to the treatment of OO at some sites difficult to reach, beyond other orthopedic conditions.

## Introduction

Osteoid osteoma (OO) is a benign bone tumor commonly occurring in the long bone, particularly in the femur and tibia. At the same time, the femoral neck is a rare site with atypical clinical manifestations. The tumor is characterized by a nidus and surrounding tissue of various degrees of ossification, with a diameter of less than 2 cm. It is composed of newly formed bone-like tissue and rich in blood vessels. Although the tumors themselves do not invade adjacent bones, they can cause bone hyperplasia and perilesional bone marrow edema, making them prone to misdiagnosis, easily confused with osteoblastoma (1). OO may heal spontaneously within 6–15 years without intervention; Oral nonsteroidal anti-inflammatory drugs (NSAIDs) can relieve the symptoms of disease; Surgical treatment is indicated in cases where medication cannot alleviate the symptoms (2). In November 2022, a child was admitted to our department with the diagnosis of OO at the femoral neck. Robot-assisted excision with intervertebral foramen endoscopy was performed due to the special site of the lesion and high risk of the surgery. The patients were evaluated clinically with the modified Harris hip score (mHHS) and visual analog scale (VAS).

## Case presentation

**Figure 1:**
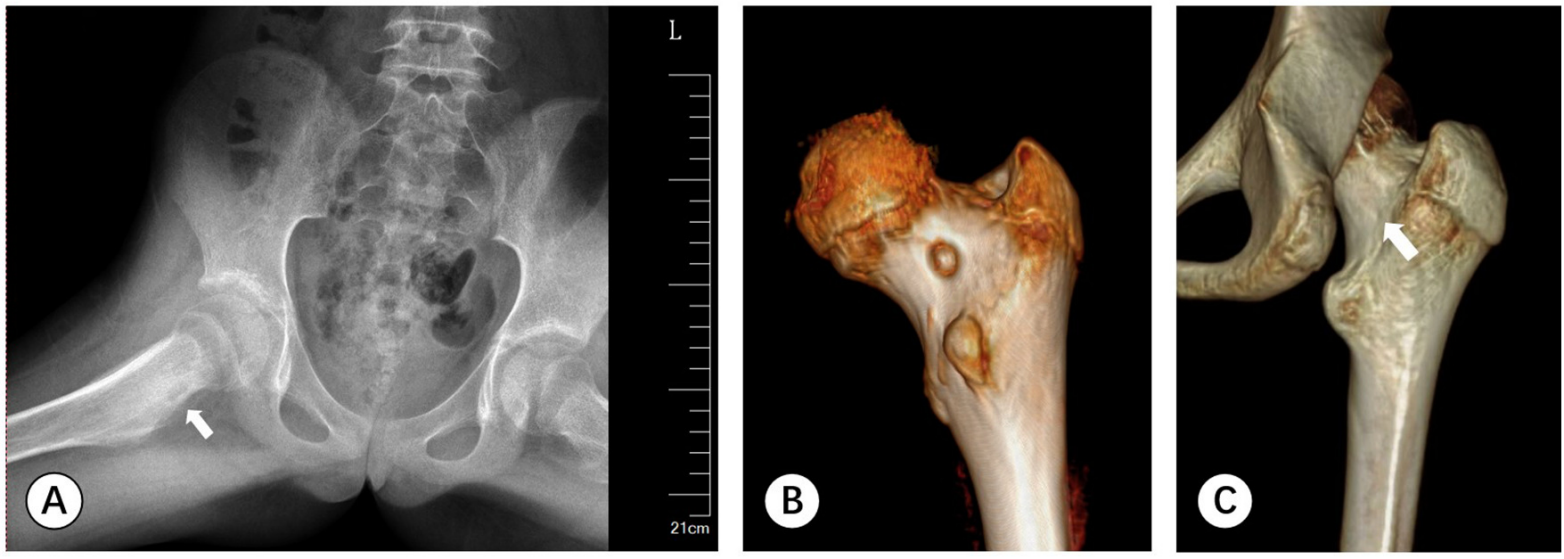
**(A)** A-P pelvic radiography shows an increase in density of the right femoral neck, with localized circular low-density shadows and slight periosteal reaction around it (white arrow). **(B)** CT scan of the posterior hip joint reveals a 7 mm circular low-density area visible on the inner side of the femoral neck. **(C)** Follow-up CT scan depicts the nidus has been completely cleared (white arrow).

An 11-year-old female was admitted to our hospital complaining of limping for the last 6 months accompanied by hip pain for more than 3 months. She had tried NSAIDs but with little success in alleviating her symptoms. The patient was referred to our hospital for further therapy. Clinical examination found limping and pseudo prolongation of the right lower limb. She had a reduced range of right hip motion, especially in flexion and rotation due to pain. Her hip radiography shows high- density cortex of the right femoral neck, with localized circular low-density shadow and slight periosteal reaction (Figure 1A). Computed tomography (CT) found a 7 mm circular low-density area on the medial side of the femoral neck (Figure 1B). MRI identified circular signal change in the medullary cavity of the proximal femur. Periosteal reaction and joint effusion also presented around the right femoral neck. With the diagnosis of OO, we performed intervertebral foramen endoscopic excision under robotic guidance six months after the patient's symptoms started.

After the operation, the patient was allowed partial weight-bearing after the first four weeks, and returned to full activity in the twelfth weeks. During this period, no recurrence symptoms appeared. The last follow-up carried out one and a half years after the operation showed that the patient was entirely pain-free (VAS score: 0) and had a full range of hip motion and normal gait (mHHS score: 100). Follow-up CT scan depicts the low-density lesion was completely erased (Figure 1C).

## Surgical procedure

The laser positioning and navigation system (Santan Medical Technology Limited Company, Hangzhou, China) used for this study was designed to provide linear guidance. The laser is assessed as class II according to the international laser classification standard (IEC 60825-1). Its wavelength is designed to be 650 ± 20 nm, and its power is 0.1 mW (3).

**Figure 2:**
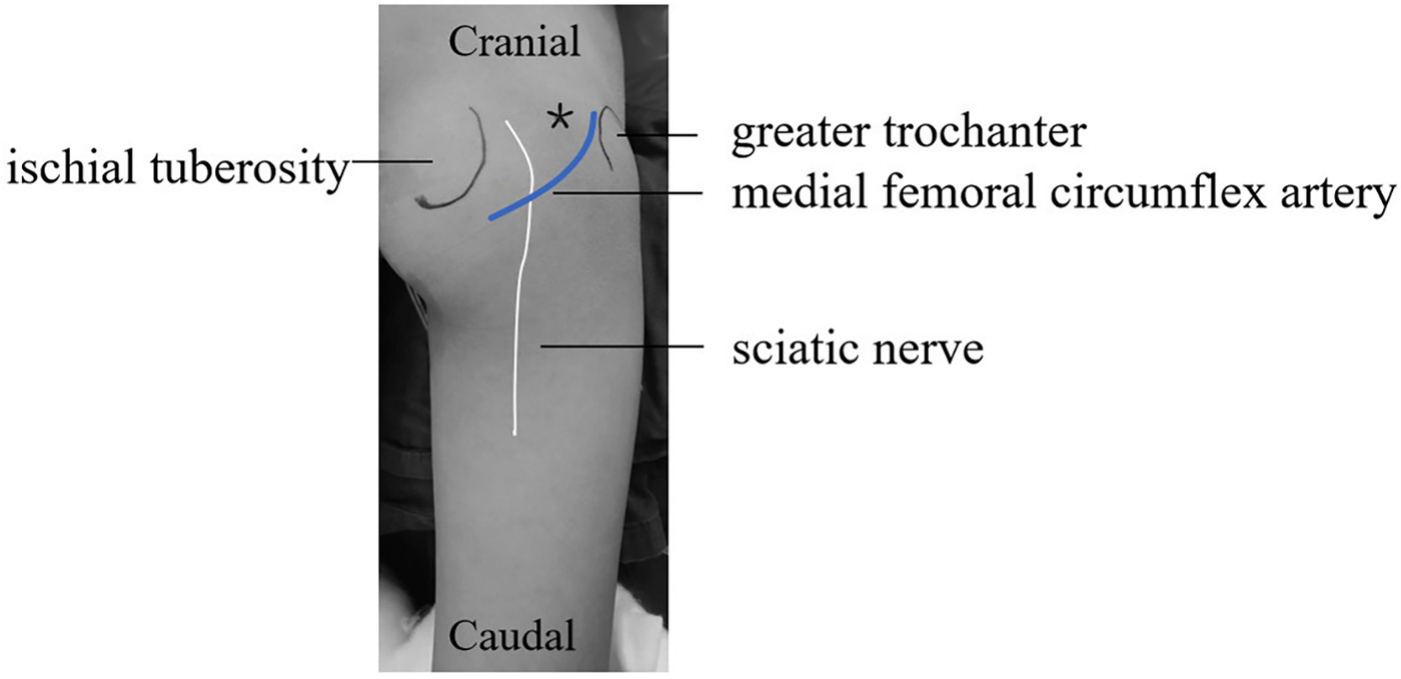
The right hip was placed in a prone position with the right lower limb was fixed in an internal rotation position. (*represent the puncture point).

**Figure 3:**
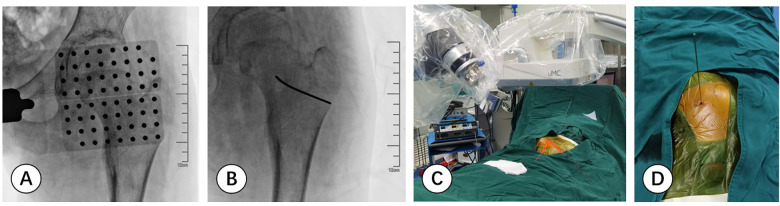
**(A,B)** fluoroscopic images are matched with the planning inserting trajectory based on scan data. **(C,D)** The surgeon precisely inserts a 1.5 mm Kirschner wire onto the edge of the lesion in the designed direction with the robotic navigation system.

**Figure 4:**
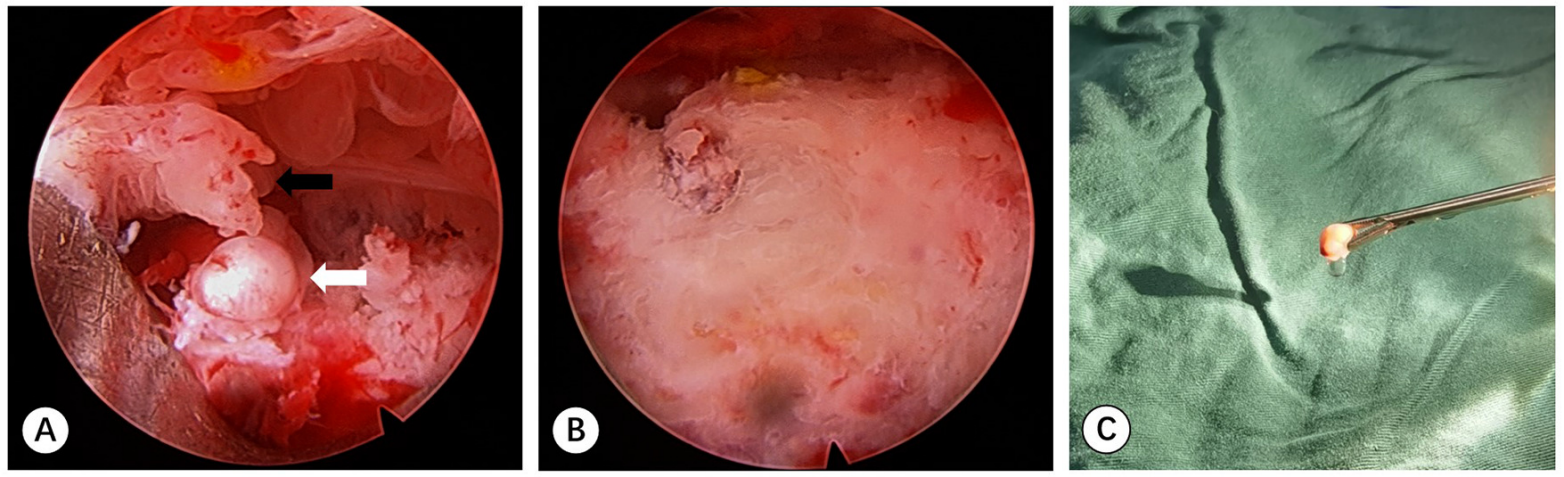
**(A)** Photograph of the endoscopy monitor demonstrating a white solid spherical nidus (white arrow) and surrounding synovial tissue (black arrow). **(B)** Endoscopic image of the tumor nest after excision of the nidus and debridement. **(C)** The oval nidus was taken out by the forceps.

Before the operation the needle trajectory was designed based on 3D-CT images, simultaneously drawing angle measurement lines in coronal, sagittal, and transverse views, avoiding the sciatic nerve and medial circumflex femoral artery. The child was placed in the prone position and her right lower limb was fixed in internal rotation (Figure 2). Surgeons installed robotic components, registered, and performed multi-modal medical image fusion. Robot-assisted navigation located the lesion, and the robot arm automatically orientated the inserting bushing. Then, a 1.5 mm Kirschner wire was directed up to the edge of the lesion without destroying the lesion (Figure 3). Intraoperative fluoroscopy confirmed the right position of the Kirschner wire. Then, a transforaminal endoscope was applied for lesion removal: a 7 mm incision was made on the skin centered at the wire, and the trocar as well as the sheath were placed along the guide wire. Remove the guide needle and clear the nearby soft tissue. A white hard spherical tumor of about 6 × 5 × 5 mm could be seen (Figure 4), and was taken out with a holding forceps. Removed the sclerotic bone around the nidus with a depth of about 2 mm under the endoscopy until the normal cancellous bone was exposed. We could observe the nidus entirely removed under endoscopic visualization directly. Finally, the surgical incision was closed with one stitch.

## Discussion

OO is a benign osteogenic tumor characterized by persistent blunt pain with nocturnal aggravation that can be relieved by oral NSAIDs. OO accounts for about 10% of benign bone tumors and most frequently affects individuals in their second and third decades of life. Osteoid osteoma is often difficult to distinguish from osteoblastoma in terms of histology (4).

At present, there are numerous treatments for OO: (1) medications: NSAIDs, bisphosphonates and salicylic acid preparation; (2) Surgical treatment: open surgical approach, CT-guided percutaneous drilling, and arthroscopic excision; (3)Radiofrequency ablation (RFA); (4) CT guided percutaneous microwave ablation (MWA) (5); (5) CT-guided percutaneous cryoablation; (6)MR-guided focused ultrasound (MRgFUS) (6). Whatever the kind of treatment adopted, the key to successful surgical treatment is accurate localization of the nidus and complete removal.

Open surgery provides a direct intraoperative vision that significantly facilitates accurate and complete removal of the lesion, improving the accuracy of pathological examination. However, the drawbacks of open surgery are also evident-inevitable local destruction that is more likely to damage adjacent neurovascular bundles and the requirement of bone grafting and/or internal fixation resulting in slower recovery (7). With RFA, there is less bleeding, shorter surgical time, and smaller bone defects. However, it has the risk of incomplete removal of the nidus and potential damage to adjacent tissues. The amount of sample collected may be too limited to make a clear pathological diagnosis. It is extremely challenging for the radiofrequency electrode needle to avoid damaging surrounding structures, such as, in this case, the sciatic nerve and the medial circumflex femoral artery. Sometimes, thermal injury could happen to the cartilage and capsule (4). Drilling may weaken the femur and induce bone necrosis which makes patients more susceptible to fractures. At present, there are reports of postoperative fractures of the femoral shaft after surgical treatment of sub-trochanteric OO, while there have been no such reports after femoral neck OO. Previous studies also reported complications such as skin burns, skin and fat necrosis, soft tissue infection, vasomotor instability, tendinitis, and hematoma (8). The clinical success rate of RFA is 94.8%, while open surgery is 90.1%. On the contrary, 4.8% of patients experience postoperative recurrence after RFA, while the rate of recurrence of open surgery is 3.7% (9). Arthroscopic excision is safe and effective, but it requires the establishment of two channels: the arthroscopic entrance and the instrument entrance.

With the development of robot-assisted navigation, in some aspects, the endoscopic spine surgery has already improved by incorporating novel technologies such as navigation, robotics, 3-dimensional (3D) and ultraresolution visualization. Compared with CT-guided RFA, the robot-assisted surgery was more efficient and accurate (10). So far, there have been no reported cases of using robot-assisted excision with intervertebral foramen endoscopy in the treatment of OO. As the lesion location in this case is rare, this innovative method can significantly improve accuracy as well as less soft tissue dissection and muscle trauma. Endoscopic applications are motivated by the clinical requirement of achieving the desired goal of treatment while minimizing trauma to the patient. Intervertebral foramen endoscopic excision only requires one small incision. It may have a longer surgical time but it reduces intraoperative bleeding and complications. A limited view of the surgical field requires extensive use of intraoperative fluoroscopy that may expose the surgeons to higher levels of ionizing radiation. With the use of robot-assisted navigation, radiation exposure is significantly reduced. Furthermore, since the navigation is planned ahead of surgery, there is less repositioning of the C-arm. In some clinical series, intraoperative fluoroscopy usage (as measured by the total fluoroscopy time) for navigation-assisted surgery is less than that for standard surgery (11). In combination, robot-assisted excision with intervertebral foramen endoscopy can provide accurate real-time information on the location, orientation, and depth of instruments. Using the ischial tuberosity and the greater trochanter as bony markers, pre-operative planning of the trajectory can avoid the sciatic nerve. It improves the accuracy of Kirschner wire insertion, allowing even inexperienced surgeons to easily insert the Kirschner wire in the correct direction. Subsequently, correct drilling trajectory and efficient nidus excision under the protection of the bushing can be achieved. Besides, surgeons may overcome the limitations induced by a narrow surgical visual field and reduce intraoperative complications such as blood loss and secondary injury (12). It can provide more meticulous management of emergency complications under intervertebral foramen endoscopy and robot navigation. These may improve the surgical outcomes of beginners and help achieve high repeatability and reproducibility. Because of the high-resolution and zoom-in effect, the control of microbleeds from bone and microcapillaries is possible, which can reduce postoperative hematomas. Additionally, this technology enables surgeons to enhance their manual dexterity with greater control and maneuverability through even a small portal, reducing physio-logical tremors. This technology still has limitations. The high cost of these instruments could impact the patient's medical costs. The learning curve of the robot is steep, and the outcome is strongly dependent on the surgeon's skills (13).

In recent years, the application of robot-assisted navigation in bone tumor surgery has become increasingly widespread. The clinical application of robot-assisted excision with intervertebral foramen endoscopy is yet uncommon, making it an issue worth exploring and promoting suitable for other orthopedic conditions.

## Conclusion

Robot-assisted excision with intervertebral foramen endoscopy was used to treat OO of the femoral neck safely and less invasively with clinical symptoms disappeared without any complication or recurrence. This combined surgical technique would be a good option under some common circumstances.

## Data Availability

The original contributions presented in the study are included in the article/Supplementary Material, further inquiries can be directed to the corresponding author.
